# FAILURE AFTER FUNDOPLICATION: RE-FUNDOPLICATION? IS THERE A ROOM FOR GASTRECTOMY? IN WHICH CLINICAL SCENARIES?

**DOI:** 10.1590/0102-672020190001e1440

**Published:** 2019-08-26

**Authors:** Italo BRAGHETTO, Attila CSENDES

**Affiliations:** 1 Department of Surgery, Hospital Clínico “Dr. José J. Aguirre”, Faculty of Medicine, University of Chile, Santiago Chile.

**Keywords:** Gastroesophageal reflux, Re-fundoplication, Barrett´s esophagus, Obesity, Distal gastrectomy, Refluxo gastroesofágico, Re-fundoplicatura, Esôfago de Barrett, Obesidade, Gastrectomia distal

## Abstract

***Background*:**

Re-fundoplication is the most often procedure performed after failed fundoplication, but re-failure is even higher.

***Aim*:**

The objectives are: a) to discuss the results of fundoplication and re-fundoplication in these cases, and b) to analyze in which clinical situation there is a room for gastrectomy after failed fundoplication.

***Method*:**

This experience includes 104 patients submitted to re-fundoplication after failure of the initial operation, 50 cases of long segment Barrett´s esophagus and 60 patients with morbid obesity, comparing the postoperative outcome in terms of clinical, endoscopic, manometric and 24h pH monitoring results.

***Results*:**

In patients with failure after initial fundoplication, redo-fundoplication shows the worst clinical results (symptoms, endoscopic esophagitis, manometry and 24 h pH monitoring). In patients with long segment Barrett´s esophagus, better results were observed after fundoplication plus Roux-en-Y distal gastrectomy and in obese patients similar results regarding symptoms, endoscopic esophagitis and 24h pH monitoring were observed after both fundoplication plus distal gastrectomy or laparoscopic resectional gastric bypass, while regarding manometry, normal LES pressure was observed only after fundoplication plus distal gastrectomy.

***Conclusion*:**

Distal gastrectomy is recommended for patients with failure after initial fundoplication, patients with long segment Barrett´s esophagus and obese patients with gastroesophageal reflux disease and Barrett´s esophagus. Despite its higher morbidity, this procedure represents an important addition to the surgical armamentarium.

## RESUMO

***Racional*:**

Re-fundoplicatura é o procedimento mais frequentemente realizado após falha na fundoplicatura, mas neste caso a falha é ainda maior.

***Objetivo*:**

a) discutir os resultados da fundoplicatura e re-fundoplicatura nesses casos; e b) analisar em que situação clínica há espaço para gastrectomia após falha na fundoplicatura.

***Método*:**

Esta experiência inclui 104 pacientes submetidos à re-fundoplicatura após falha da operação inicial, sendo 50 casos de esôfago de Barrett de segmento longo e 60 pacientes com obesidade mórbida, comparando-se o resultado pós-operatório em termos de pH clínico, endoscópico, manométrico de 24 h de monitoramento.

Em pacientes com falha após a fundoplicatura inicial, a re-fundoplicatura mostra os piores resultados clínicos (sintomas, esofagite endoscópica, manometria e pHmetria 24 h). Em pacientes com esôfago de Barrett de segmento longo, melhores resultados foram observados após fundoplicatura com gastrectomia distal em Y-de-Roux e em pacientes obesos resultados semelhantes em relação aos sintomas, esofagite endoscópica e monitoramento de pH 24 h foram observados após fundoplicatura com gastrectomia distal ou ressecção com bypass gástrico laparoscópico, enquanto que em relação à manometria, a pressão normal do EEI só foi observada após a fundoplicatura e gastrectomia distal.

***Conclusão*:**

A gastrectomia distal é recomendada para pacientes com falha após a fundoplicatura inicial, pacientes com esôfago de Barrett de segmento longo e obesos com doença do refluxo gastroesofágico e esôfago de Barrett. Apesar de sua maior morbidade, esse procedimento representa um importante acréscimo ao arsenal cirúrgico.

## INTRODUCTION

Total or partial fundoplication, are the current surgical treatments for gastroesophageal reflux disease (GERD). However, there are circumstances in which the procedure fails due to an incorrect or incomplete preoperative evaluation, technical factors or the patients´ characteristics. Therefore, it is necessary to find the best technique in order to assure the best long term outcome after this operation.

There are three clinical situations in which fundoplication fails: 1) recurrent disease after failed fundoplication; 2) in Barrett´s esophagus; 3) in obese.

The mean reported failure after fundoplication is nearly 18% (3-33%) associated with recurrent reflux symptoms or erosive esophagitis, and 4.5-20% of these patients need re-operation^5^ ^,^ ^20^ ^,^ ^21^ ^,^ ^29^ ^,^ ^51^ ^,^ ^52^. The reported failure in non-Barrett´s patients is 5-12% and in Barrett´s failure is observed three times more frequently, from 12 to 39%. The recurrence of symptoms due to failed fundoplication reaches 31.3% in obese^24^. Patients with BE have more incompetent lower esophageal sphincter (LES), severe anatomic distortions, greater amount of acid and bile reflux and severe damage of distal esophagus. Therefore, fundoplication becomes more difficult and complex and it is associated with a high rate of failure. 

The main reasons for failure are esophageal dysmotility and delayed clearance associated with physiologic alterations of the LES. Other reasons are secondary to the surgical technique performed such as the absence of clear anatomical landmarks, anatomical deformities due to dilated cardia, presence of a hiatal hernia or complicated long segment Barrett´s esophagus (LSBE) or obesity. These can provoke surgical difficulties during the procedure conducing to abnormal primary repair or occurrence of disruption, slippage, mal position or herniation, associated with postoperative abnormal antireflux or defective valve as causes for reoperation^6^. 

The objectives of this article are: a) to discuss the results of fundoplication and re-fundoplication in these cases, and b) to analyze in which clinical situation there is a room for gastrectomy after failed fundoplication. 

## METHOD

Our personal experience in 104 patients with failure after fundoplication and submitted to re-fundoplication or distal gastrectomy (RYDG) is presented. Also the experience in 50 cases with long segment Barrett´s esophagus and in 60 obese submitted to fundoplication plus Roux-en-Y distal gastrectomy (RYDG) or laparoscopic resectional Roux-en-Y gastric bypass (LRGBP) alone, are included in order to compare the postoperative outcome after these procedures in terms of clinical( symptoms), endoscopic evaluation, manometry in order to evaluate lower esophageal sphincter (LES) pressure and 24h pH monitoring postoperative results. 

## RESULTS

### After re-fundoplication

Table 1 shows our results observed in 104 patients with failure after initial fundoplication. Symptoms after surgical treatment for recurrent postoperative gastroesophageal reflux submitted to re-fundoplications alone vs. re-fundoplication combined with Roux-en-Y distal gastrectomy is shown. After re-do fundoplication, almost 20% of the patients continued having symptoms while symptoms were significantly less after re-fundoplication with Roux-en-Y distal gastrectomy (acid suppression/duodenal diversion surgery), in which only 5.5% of the patients presented reflux symptoms (p<001). In Table 2 the results concerning to endoscopy, manometry, acid and bile reflux test before and after these procedures are shown. Recurrence of esophagitis after redo surgery alone was observed in 23.5% of the patients, which was significantly greater compared to the endoscopic findings of esophagitis observed in patients submitted to distal gastrectomy (p<0.001). In patients with complicated Barrett´s esophagus (with ulcer or strictures), endoscopic recurrence of erosive esophagitis was present in 100% of the patients operated upon with redo fundoplication alone; however, only 4.8% of patients with Roux-en-Y distal gastrectomy presented recurrence of esophagitis. Preoperative acid reflux was present in 94.7% of patients, in 93.6% after initial fundoplication and in 68.8% after redo-fundoplication. On the contrary, acid reflux was only present in 16.6% of patients submitted to distal gastrectomy (p< 0.001, Table 2). These results suggest that re-fundoplication is not a good option.

**TABLE 1:** Symptoms after surgical treatment for recurrent postoperative gastroesophageal reflux comparing re-fundoplications vs. Roux-en-Y distal gastrectomy (RYDG) (n=104)

Symptoms	First operation		After re-operation	
	Preop	Posop	Re-fundoplication	RYDG
Heartburn	100%	100%	19.2%	5.5% (p<0.05)
Regurgitation	86.7%	80.6%	11.5%	2.7% (p<0.05)
Chest pain	29.5%	28.5%	3.8%	-
Anemia	6.1%	9.1%	-	-
Resp. symptoms	5.1%	3.0%	-	-

**TABLE 2:** Endoscopy, manometry, acid and bile reflux before, after operation and after the reoperation (n=104)

	First operation		After re-operation	
	Preop	Posop	Re-fundoplication	RYDG
Erosive esophagitis	100%	83.4%	23.5%	7.7%*
Barrett with esophagitis				
Ulcer/stricture	100%	100%	100%	4.8%*
Incompetent LES	82%	56%	53%	32%
Positive acid reflux	95%	94.3%	69%	11.1%*
Biliary reflux	-	57.6%	40%	0

*p<0.001

### Barrett´s esophagus

Table 3 shows our experience in 50 patients with Barrett´s esophagus submitted to laparoscopic fundoplication alone or combined with distal gastrectomy procedure. Symptoms recurrence, radiologic failures demonstrating positive reflux and inadequate antireflux valve, endoscopy demonstrating erosive esophagitis with Barrett´s esophagus, persistence of positive reflux test or defective manometry after surgical treatment were significantly less frequent when combination of fundoplication plus distal gastrectomy was performed. This later operation presents low rate of major complications (Table 4).

**TABLE 3:** Symptomatic and objective failures after laparoscopic surgery in patients with Barrett´s esophagus submitted to fundoplication alone vs. combined fundoplication plus Roux-en-Y distal gastrectomy (RYDG) (n=50)

	Long Segment Barrett´s esophagus	
	Fundoplication	Fundoplication+RYDG
	(n= 22)	(n=28)
Symptoms recurrence	36.5%	3.7%
Radiologic failure	27.3%	3.7%
Endoscopic failure	50%	3.7%
Positive acid reflux	40.9%	3.7%
Incompetent LES	31.8%	21.4%

**TABLE 4:** Early and late outcome after laparoscopic Roux-en-Y distal gastrectomy

	Mean	Range
Complications	30.9%	21-46%
Re-operations	11%	9-12.5%
Mortality	0	
Hospital stay (days)	6	1-33
Satisfaction rate	92%	88-96%

References: ^10^ ^,^ ^33^ ^,^ ^37^ ^,^ ^45^ ^,^ ^49^

### In obese patients 

In Table 5 and 6 the experience in 60 obese patients with Barrett´s esophagus with BMI <30 kg/m^2^ or >30 kg/m^2^ is shown. The best results were observed after fundoplication Roux-en-Y distal gastrectomy (RYDG) or laparoscopic resectional Roux-en-Y gastric bypass (LRGBP) alone in LSBE patients in terms of symptoms, endoscopic findings, manometric and acid reflux tests. 

**TABLE 5:** Clinical results after fundoplication plus Roux-en-Y distal gastrectomy (RYDG) and laparoscopic resectional gastric bypass ( LRGBP) in patients with long segment Barrett´s esophagus (n=60)

	LSBE		
	Fundoplication+RYDG	LRGBP	
Reflux Symptoms			
Preop	39 (100%)	21(100%)	
Postop	1 (2.6 %)	0	(p=0.45)
Erosive esophagitis			
Preop	39 (100%)	21(100%)	
Postop	1 (2.6 %)	0	(p=0.45)
Esophageal ulcer/stricture			
Preop	4 (10.2 %)	6(28.6%)	
Postop	0	0	
Histology (presence of intestinal metaplasia)			
Preop	39 (100%)	21 (100%)	
Regression	20 (51.3%)	13 (61.9%)	(p=0.35)

**TABLE 6:** Manometry and 24h pH monitoring after Fundoplication plus RYDG and LRGBP in patients with long segment Barrett´s esophagus (n=60)

	LSBE	
	Fundoplicatio+RYDG	LRGBP
Hipotensive LES pressure		
Preop	39(100%)	21(100%)
Postop	5 (12,8%)	21(100%)
Abnormal acid reflux		
Preop	39(100%)	21(100%)
Postop	1 (2,6%)	2(9,5%)

## DISCUSSION

First of all, the decision to indicate reoperation after failed fundoplication is not easy. The decision to choose a remedial antireflux procedure is a challenging clinical problem which must be individualized depending on the clinical features of the patients, the characteristics and severity of symptoms, type of esophagitis, presence of ulcer, stricture, Barrett´s esophagus, delayed gastric emptying, acid/bile reflux, the number of previous operations and the presence of obesity^10^ ^,^ ^12^ ^,^ ^24^ ^,^ ^27^ ^,^ ^35^ ^,^ ^38^ ^,^ ^44^. All of these factors must be taken into account in order to decide which surgery is the best option after a failed fundoplication. 

To better understand this issue, the authors made a complete revision of the literature looking for the terms “failed fundoplication”, “reoperation after fundoplication”, “results after fundoplication”, “surgery for Barrett´s esophagus”, ”antireflux surgery in obese patients”. For the data collection the searched bases were Pubmed, Medline, Cochrane library, Scopus, Google Scholar in order to have a complete vision of the problem. A total of 52 papers were reviewed to obtain the most accurate data regarding what the options are for treating a failed fundoplication.

The alternatives to treat failure are: 

### Redo fundoplication

This is the most frequent procedure used as a first approach. It is used in 89% of cases. It is a demanding procedure with a longer operative time, and it is more complex and difficult to perform compared to the first fundoplication. It can frequently present intra-operative complications such as bleeding, perforation, and spleen injury, which are more common compared to the initial fundoplication and increase after the first or second re-operation. The rate of intra-operative complications is 17-22% for the first re-operation and it reaches 36% during the second. The postoperative complications range from 15-27% and a long hospital stay may be required (6-58 days). The success rate after the first re-fundoplication is very variable ranging from 42 to 94% and decreases to 60% after the second re-fundoplication^3^ ^,^ ^7^ ^,^ ^20^ ^,^ ^23^ ^,^ ^25^ ^,^ ^28^ ^,^ ^34^ ^,^ ^35^ ^,^ ^45^ ^,^ ^46^ ^,^ ^51^ ^,^ ^52^ ^,^ ^53^. In addition, studies addressing long term outcomes have observed 27 to 41% of reflux recurrence with incomplete relief of symptoms in 12-50% of patients submitted to re-fundoplication. A very low satisfaction rate has been also reported with less than 50% of patients manifesting satisfaction after the re-operation^49^. Failure after the first redo-fundoplication is 12-20% and reaches 40% in cases submitted to a second redo. The worst results have been reported in obese or Barrett´s esophagus patients^7^ ^,^ ^20^ ^,^ ^25^ ^,^ ^41^ ^,^ ^49^ ^,^ ^50^ ^,^ ^51^. 

Fundoplication combined with RYDG or LRGBP alone 

Re-fundoplication plus RYDG is performed in only 12% of patients in North America. This is a more complex procedure because it involves two steps: first to perform re-fundoplication itself and second a distal gastrectomy. On the contrary, in patients with failure of initial fundoplication, or LRGBP alone without redo fundoplication avoids the performance of surgical dissection on an unfavorable surgical field with unclear anatomy due to the adhesions, fibrotic and distorted tissues. However, it is an incomplete procedure because it does not fix the inadequate anti-reflux barrier due the prior disrupted, slipped, asymmetric or herniated fundoplication. Therefore, it is possible to postulate that the combination of both procedures could be the best choice in order to avoid acid and bile reflux. The disadvantage of this alternative is the high rate of in-hospital or postoperative morbidity which can reach up to 67% after gastrectomy compared to only 20% after refundoplication (p=0.007)^10^ ^,^ ^12^ ^,^ ^31^ ^,^ ^33^ ^,^ ^37^ ^,^ ^45^ ^,^ ^47^ ^,^ ^49^ ^,^ ^51^. On the other hand, the advantage of distal gastrectomy is relief of reflux symptoms after surgery, which reach nearly 89% compared to 50% after refundoplication (p=0.044). In selected patients with severe GERD, multiple previous fundoplications, other clinical situations such as Barrett´s esophagus or obesity, we believe that gastrectomy is an acceptable treatment option with a significantly better long term outcome^15^ ^,^ ^30^ ^,^ ^35^ ^,^ ^40^. 

Concerning to the experience with long segment Barrett´s esophagus (LSBE), the definitive treatment for patients with it remains controversial. For gastroenterologists, medical treatment with PPIs combined with ablation of Barrett´s mucosa has demonstrated excellent long-term follow-up in terms of symptoms control and regression of metaplasia and even dysplasia^17^ ^,^ ^22^ ^,^ ^27^ ^,^ ^28^ ^,^ ^29^ ^,^ ^42^ ^,^ ^47^ ^,^ ^49^ ^,^ ^53^. Surgeons believe that laparoscopic anti-reflux surgery presents nil morbimortality and also very good results. The combination of fundoplication with ablation is a new way to explore^27^ ^,^ ^31^. However, long term follow after fundoplication alone in BE patients is unsatisfactory because acid and bile reflux persist in a high proportion of patients as we commented before^4^ ^,^ ^8^ ^,^ ^14^ ^,^ ^15^ ^,^ ^16^ ^,^ ^22^.

The goals of surgical treatment, are: a) controlling symptoms of gastroesophageal reflux disease; b) abolishing acid and duodenal reflux into the esophagus; c) preventing or eliminating the development of complications; d) preventing extension of or an increase in the length of the intestinal metaplasia; e) inducing regression of the intestinal metaplasia to the cardiac mucosa; and f) preventing progression to dysplasia, thereby inducing regression of low-grade dysplasia and avoiding the appearance of adenocarcinoma.

Experiences reported in patients with LSBE submitted to fundoplication have demonstrated unsatisfactory results at long term follow-up. Many authors have reported poor results due to a high rate of symptoms recurrence. Acid reflux persists at 8-10 years after surgery in a high proportion ranging between 7%-60% of patients. Duodenal reflux is present in 95% of them, and peptic ulcer, stricture, and erosive esophagitis appears in 15-30% late after surgery. Bowers and Oeschaleger^4,42^ have reported regression of intestinal metaplasia in 33% and 55% of patients respectively, and regression of low-grade dysplasia in 45% of patients with short segment Barrett´s esophagus but not in patients with LSBE. Progression of the disease has been observed in almost 50% of patients with appearance of low-grade dysplasia in 6.0% and adenocarcinoma in 3.4%^9^ ^,^ ^17^ ^,^ ^18^ ^,^ ^28^.

Therefore, in patients with LSBE, the clinical results after fundoplication are not optimal. No long-lasting effect has been demonstrated and it does not prevent the appearance of dysplasia or adenocarcinoma. On the contrary, progression to dysplasia was observed in nearly 18% of cases at 5-years follow-up^9^. 

Therefore RYDG is based on the following points: a) better control of severe gastroesophageal acid reflux and frequent duodenoesophageal reflux; b) better late results compared to the classic anti-reflux procedure in BE; c) better healing of the esophageal damage produced by the injurious component of the refluxate. The authors have observed that the simple correction of the valve is not enough in many cases because it does not abolish the gastroesophageal reflux but only diminishes it. In patients who have BE and therefore have impaired esophageal clearance, few reflux episodes can maintain or even induce more damage. With the acid suppression/duodenal diversion procedure, the quality of the corrected valve is secondary, and the main goal is to avoid the reflux of injurious components of the refluxate instead of the refluxate itself which is almost always impossible. 

Late results support this hypothesis and this surgical procedure as an alternative treatment in patients who have complicated BE or in patients who have long-segment BE. 

Laparoscopic Roux-en-Y distal gastrectomy (RYDG) is an excellent alternative which has demostrated 91% clinical success for more than five years. This procedure has almost eliminated acid and duodenal reflux, and there has been no progression to dysplasia or adenocarcinoma. Moreover, in 60% of the patients with low-grade dysplasia, regression to non-dysplastic mucosa has occurred^8^ ^,^ ^15^ ^,^ ^16^. 

The disadvantage of this operation is the high rate of complications and longer stay in hospital. However, no mortality has been reported after this technique and for us and many other authors the most important point is the very satisfactory results at long time follow-up. These results suggest that distal gastrectomy, in spite of having higher morbidity, is an acceptable treatment option with better long term outcome^25^ ^,^ ^31^ ^,^ ^33^ ^,^ ^37^ ^,^ ^47^ (Table 4).

### The experience in obese patients

In obese patients with GERD or Barrett´s esophagus there are two problems: a) reflux and its complications, and b) obesity. 

In order to obtain an improvement of reflux and obesity, patients must be submitted to surgical procedures in order to accomplish both purposes: treat reflux (minimizing recurrences) and improve obesity index.

Then, the surgical options for treatment are: 

#### *Fundoplication alone*

The results are not as good as in patients who are not severely obese and besides does not treat obesity. Morgenthal^39^ demonstrated that one of the important factors for recurrence of GERD after laparoscopic Nissen fundoplication is obesity, and a preoperative morbid obesity (BMI>35 kg/m^2^) was clearly associated with failure (p=0.036). High rate of recurrence of gastroesophageal reflux has been demonstrated after fundoplication^36^ ^-^ ^38^. In our own experience, we reported almost 50% of reflux after fundoplication alone in obese patients^10^. Perez found that recurrence after fundoplication is correlated with increased BMI; so, in normal weight the recurrence is 4.5%, 8% in overweight and 31% in obese patients^45^.

#### *Combined procedures or gastric bypass with resection*

In our experience, obese patients with BMI >30 with LSBE, two procedures have been employed: fundoplication+RYDG and LRGBP^10^. The main surgical differences between these two procedures are: a) addition of fundoplication in the former procedure; b) greater resection of the stomach (95%), leaving a small gastric pouch of 30 ml of capacity after LRGBP while the resection of distal stomach is approximately 40% after Fundolication+RYDG; c) the length of the alimentary Roux-en-Y limb is 60-70 cm after RYDG while after LRGBP it is closer to 130-150 cm.

The indication of one procedure or other depends on the basal BMI and presence of co-morbidities. So, for non-diabetic or non-dislipidemic with BMI 30-35 we offer Fundoplication+RYDG. On the contrary, diabetic or dislipidemic patients with BMI more than 35 were indicated LRGBP in order to treat all these co-morbidities and reflux disease.

 Despite the initial controversy or rejection of this idea by the majority of surgeons dedicated to this subject, in recent years it has been accepted due to the benefits of a long term follow-up. Others authors have confirmed a decrease in reflux symptoms, proton pump medication, acid reflux and complete regression of Barrett´s esophagus after LRYGBP and for them, LRYGBP is the preferred surgery in morbidly obese patients with BE^12^ ^,^ ^19^ ^,^ ^30^ ^,^ ^32^ ^,^ ^37^. This concept confirmed our idea suggesting this technique as an excellent strategy for these patients^19^. 

Recent papers concluded that laparoscopic conversion of Nissen fundoplication to Roux-en-Y gastric bypass is a technically feasible and safe operation for recurrent gastroesophageal reflux disease in the morbidly obese with prior anti-reflux surgery, and in obese patients requiring surgical treatment for gastroesophageal reflux disease^1^ ^,^ ^12^ ^,^ ^19^ ^,^ ^30^ ^,^ ^31^. In our opinion, laparoscopic Roux-en-Y gastric bypass is the best option for morbidly obese patients with Barrett´s esophagus. In addition, in a recent publication, Altieri^1^ concluded that Roux-en-Y gastric bypass is associated with a decreased incidence of GERD and it is the procedure of choice for obese with GERD and for patients with Barrett´s esophagus^2,11,26,28,37,39,40,45 46,52^.

## CONCLUSION

Patients who have BE have anatomical and physiological foregut abnormalities which cannot be improved with anti-reflux surgery or reoperations. However, laparoscopic re-fundoplications continue to be the most common revisional surgery after failed fundoplication, but the late results are poor and with a high recurrence rate. Barrett´s patients also have a high rate of recurrence and worse so after re-fundoplication. There are patients that have been unnecessarily submitted to 1, 2 or even 3 re-operations. In these cases, fundoplication plus acid suppression/duodenal diversion techniques with Roux-en-Y distal gastrectomy provides better control of the disease. Therefore, there is a place for this procedure in patients with failure after initial fundoplication because it is adequate, safe and reliable as a first line therapy. 

In obese with gastroesophageal reflux disease and Barrett´esophagus an acid suppression/bile diversion procedure similar to gastric bypass has been recommended as an effective combined bariatric and anti-reflux surgical procedure, achieving endoscopic and histologic regression to normal mucosa in a substantial number of patients.

Despite its higher morbidity, this procedure represents an important addition to the surgical armamentarium. For us, it is the first option in these three clinical situations.
